# Convergent Evolution and the Epigenome

**DOI:** 10.3390/epigenomes9040045

**Published:** 2025-11-11

**Authors:** Sebastian Gaston Alvarado, Annaliese Chang, Maral Tajerian

**Affiliations:** 1 Department of Biology, Queens College, City University of New York, 65-30 Kissena Blvd, Flushing, NY 11361, USAmaral.tajerian@qc.cuny.edu (M.T.); 2 Graduate Center, City University of New York, 365 5th Ave, New York, NY 10016, USA

**Keywords:** convergent evolution, epigenetic regulation, phenotypic plasticity, DNA methylation, PRDM9, recombination hotspots, nucleosome occupancy, mutation bias, regulatory evolution

## Abstract

Background: Trait convergence or parallelism is widely seen across the animal and plant kingdoms. For example, the evolution of eyes in cephalopods and vertebrate lineages, wings in bats and insects, or shark and dolphin body shapes are examples of convergent evolution. Such traits develop as a function of environmental pressures or opportunities that lead to similar outcomes despite the independent origins of underlying tissues, cells, and gene transcriptional patterns. Our current understanding of the molecular processes underlying these phenomena is gene-centric and focuses on how convergence involves the recruitment of novel genes, the recombination of gene products, and the duplication and divergence of genetic substrates. Scope: Despite the independent origins of a given trait, these model organisms still possess some form of epigenetic processes conserved in eukaryotes that mediate gene-by-environment interactions. These traits evolve under similar environmental pressures, so attention should be given to plastic molecular processes that shape gene function along these evolutionary paths. Key Mechanisms: Here, we propose that epigenetic processes such as histone-modifying machinery are essential in mediating the dialog between environment and gene function, leading to trait convergence across disparate lineages. We propose that epigenetic modifications not only mediate gene-by-environment interactions but also bias the distribution of de novo mutations and recombination, thereby channeling evolutionary trajectories toward convergence. An inclusive view of the epigenetic landscape may provide a parsimonious understanding of trait evolution.

## 1. Introduction

### 1.1. Genetic Convergence as a Parsimonious Explanation for Trait Convergence

The phenomenon of trait convergence has been widely reported across the plant and animal kingdoms. It describes the evolution of similar traits across a continuum of homologous (parallelism) and non-homologous (convergent) substrates subject to similar environmental pressures [1]. This continuum can be illustrated by the evolution of wings in bats, birds, and insects. While wings serve a similar function in permitting flight, the forelimb origins of wings in bats and birds sit closer on the scale of parallelism than the origins of wings from gills seen in insects [2]. These examples are countless and have even been documented across biological scales (from the evolution of antifreeze proteins in arctic/antarctic fishes [3] to the carcinization of body plans in crustaceans [4]). In this review, we focus on three well-characterized epigenetic processes to illustrate how molecular plasticity can bias evolutionary outcomes. These should be viewed as representative examples within a much broader landscape of mechanisms that also generate biological variation. Our aim is not to provide an exhaustive catalogue, but rather to highlight a curated set of processes that exemplify how epigenetic regulation may repeatedly channel evolution toward convergent solutions.

**Figure 1:**
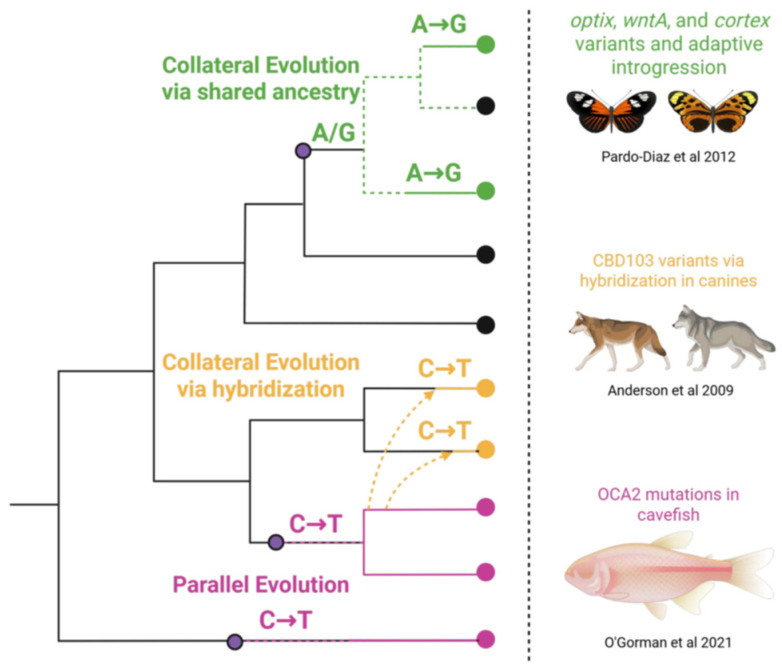
Overview of genetic processes that contribute to convergence across lineages. Parallel evolution occurs when similar mutations arise independently in the same gene across lineages (pink). Collateral evolution results from shared ancestry (green) or hybridization (yellow) along with examples from the literature of each [11,12,13].

Due to the broad comparative scope of these phenomena, the underlying mechanisms at the molecular level must also be well-conserved in function. Genes have long been viewed as mechanistic substrates of many biological traits; however, convergent traits challenge how conserved heritable substrates have independent origins. The mechanistic processes driving convergence have been consistently studied from the “bottom-up”, focusing primarily on genes, the cells that express them, and the traits they underlie. The genetic causes of convergent evolution are partly caused by parallel and/or collateral evolution through shared ancestry/hybridization [5] (Figure 1). Parallel evolution describes how similar mutations in the same genes occur across distantly related lineages. For example, resistance to plant neurotoxins has been reported in herbivorous insects through independent non-synonymous mutations of the GABA receptor [6]. In the collateral evolution, shared ancestry or introgression contributes to alleles of major effects as a function of natural selection. For example, in *Heliconius* subspecies, ancestral cis-regulatory loci located near rays and dennis patterning genes have become fixed in certain subpopulations, entire populations, and hybrid zones within the Amazon, providing evidence for both forms of collateral evolution [7]. Introgression is often cited as a source of genetic variation within closely related groups, as shown in wheat ears [8], mussels [9], and whitefish [10]. It is important to note that these processes rely entirely on the assumptions of standard evolutionary theory and the neutral theory of molecular evolution. That is, genetic variation happens by chance, and convergence happens via the selection of alleles of major effect. In this review, however, our primary focus is on the circumstances that predispose certain genomic regions to parallelism. Rather than arising purely from stochastic drift followed by selection, we consider how epigenomic features and chromatin state may actively bias the frequency and location of where mutations occur, producing predictable and repeatable evolutionary outcomes.

Recent decades have framed the modern evolutionary synthesis and the neutral theory of evolution as the primary frameworks for explaining convergence. Briefly, the evolutionary synthesis posits that adaptive traits arise through the gradual accumulation of genetic variation shaped by natural selection, while the neutral theory emphasizes that most genetic changes are selectively neutral and that variation arises largely through random mutation and genetic drift. However, both frameworks present conceptual challenges regarding how convergence can function under these genetic mechanisms and have remained under scrutiny for the last half-century [14]. Under the neutral theory, the likelihood of molecular convergence is low, and the probability decreases with increasing genetic distance between lineages [15]. This raises additional questions about how shared environments shape biology in predictable patterns via environment-by-gene interactions and phenotypic plasticity. Such predictability has prompted new considerations for the role of environmental context in shaping biological “chance”, explored through ecological-developmental [16], plasticity-first [17], and transgenerational epigenetics [18] perspectives. Beyond these classical models, additional frameworks emphasize how genomes may actively restructure themselves. For instance, Shapiro and colleagues have advanced the concept of “natural genetic engineering”, [19] in which mobile elements, recombination machinery, and epigenetic regulators function within a flexible, read/write genome architecture. While our review is focused on three well-characterized epigenetic processes, these perspectives highlight that mutational processes are not entirely blind but can be biased or facilitated by other molecular mechanisms [20,21], complementing our thesis that epigenetic regulation channels evolutionary outcomes.

### 1.2. Epigenetic Priming of Convergent Evolution

A gene-centric interpretation of trait convergence is tractable in closely related lineages, particularly when it involves hybridization or shared ancestry. However, many striking examples of convergence, especially among distantly related taxa, arise from independent de novo mutations. This suggests that molecular evolution is not purely stochastic or limited by lineage. For instance, the streamlined body plans of cetaceans, penguins, and fish, despite vast phylogenetic distances, are unlikely to result from shared genetic ancestry alone. In such cases, selection imposed by a common environment likely channels variation toward similar adaptive outcomes. Phenotypic plasticity, the capacity of a single genotype to produce multiple phenotypes in response to environmental cues, introduces an evolvable layer of flexibility that may bias evolutionary trajectories [22]. Plasticity allows organisms to shift between traits suited for changing environments, generating cryptic variation that may later become genetically assimilated [23]. These transitions have been proposed as mechanisms by which environmentally induced traits become fixed over time [17]. This relationship between plasticity and evolution is supported by empirical evidence. In threespine stickleback, for example, genes showing strong gene-by-environment expression plasticity are also frequent targets of recurrent mutation during parallel adaptation to freshwater environments [24]. This suggests that the genome is not a blank slate, but a reactive system shaped by feedback between plastic responses and mutational inputs.

In distantly related lineages, where shared homology cannot explain convergence, environmentally responsive molecular substrates may influence the location and frequency of de novo mutations. These biases could arise from features like chromatin accessibility, DNA methylation, transcriptional activity, or replication timing, all of which vary with environmental or developmental context [25,26,27]. Whether in closely related species or deeply divergent taxa, the recurrence of similar phenotypes suggests underlying molecular regularities that are neither wholly random nor strictly genetically predetermined. When phenotypic convergence spans large evolutionary distances, traditional gene-centric models often fail to provide a complete explanation. This invites a broader framework in which plastic molecular substrates, especially those sensitive to environmental input, mediate where and how mutation, selection, and constraint operate over time. The repeated involvement of highly conserved, environmentally responsive pathways, such as chromatin remodeling complexes or DNA methylation machinery, suggests that evolution may repeatedly draw on the same molecular mechanisms to solve similar ecological challenges. In the sections that follow, we explore how such molecular plasticity may leave lasting signatures on the genome and help explain both the predictability and repeatability of evolution.

### 1.3. Bias in De Novo Mutations and Recombination

Mutational variation across the genomic landscape provides an essential source of natural variation that forms the basis of the neutral theory of molecular evolution. However, emerging evidence suggests that these processes are targeted in alleles of major effect [28] and may not be random. While this variation can arise from many different sources such as nucleotide chemistry [29] or recombination, the mutational landscape demonstrates bias and plasticity. For example, it has been suggested that paternal age increases the mutational load as a function of spermatogenesis production [30,31]. However, this increase in mutational frequency has also been implicated in de novo mutations underlying autism spectrum disorders [32,33], suggesting nonrandom effects on behavioral traits. While paternal age effects have been broadly documented in mammals [34,35], recent evidence suggests other biases may exist across the kingdom of life. Even comparative analyses on male mutational bias have shown that this bias cannot be entirely attributed to spermatogenesis divisions [36,37], and are also present (to a lesser degree) in females [38,39].

Various diseases have been attributed to de novo mutational biases in human populations. Exome sequencing in ALS patients has shown that amino acid-altering mutations were enriched in genes encoding chromatin regulators (nBAF and CREST) that functionally contribute to disease ontology [40]. Similarly, in autism spectrum disorders, Samocha et al. showed that loss-of-function mutations were under strong evolutionary selection in complex diseases [41]. While these examples are not themselves adaptive, they provide some of the most data-rich demonstrations that mutational processes are biased toward particular loci, revealing that such events do not occur blindly across the genome. We highlight them here as illustrative cases because of the richness of available datasets, which make the underlying biases observable in ways that are difficult to capture in adaptive systems. This empirical foundation complements adaptive cases, which more directly connect mutational bias to convergent outcomes.

However, recent reports in human populations have shown that de novo mutation rates target biologically relevant genes in human sperm [42] based on biogeography and their natural selective pressures. Using sperm collected from male donors living in Europe and Africa, Melamed et al. showed that de novo sickle cell mutations within HBB occurred uniquely among African men compared to European controls. These changes were restricted within the HBB gene and not in paralogs in the same individual. In a separate study on the same populations, de novo resistance mutations to Trypanosoma were also found for African individuals compared to European controls [43]. These data suggest that selective pressures (malaria and sleeping sickness) can guide a population to acquire specific mutations that give them a selective advantage in their environment. These phenomena are not restricted to placental mammals. In the Andean house wren, paralogs of the globin gene (HBA1) are targeted for mutagenesis with environmental pressure of altitude [44], thus affecting the oxygen-carrying capacity of their blood cells. These adaptive cases provide clearer examples of how environmental forces can channel mutational outcomes toward convergent traits. These outcomes have been used to support the broader idea of “evolutionary honing” that considers environmental pressures that may shape the incidence of mutations in alleles of significant effect [45]. We suggest that these biases can be scaled and attributed to recombination hotspots seen in eukaryotic genomes.

Recombination hotspots introduce bias via the uneven distribution of genetic variation, shaping genomic architecture, function, and evolution [46,47]. In a comprehensive resequencing of an Avian pedigree, recombination hotspots were shown to be situated within genic regions, with the highest density in promoters and CpG islands [48]. These findings were also replicated in dogs [49] and snakes [50], suggesting that cis-regulatory regions are targets for recombination hotspot-mediated mutational bias. The resulting genetic structure can influence allele frequencies through gene conversion, which is biased toward GC-rich alleles due to the selective advantages they might confer [51]. While recombination hot spots play an evident role in the mutational landscape, natural environmental triggers can shape the formation of recombination hot spots. For example, in barley and rice, recombination rates increased in genic regions that appeared to be involved in responses to environmental stimuli [46,52]. These recombination hot spots tend to show less genetic load and lower accumulation of deleterious mutations [53]. In vertebrates, environmental shifts in temperature [54,55,56] and population demography [57] can also shape the distribution of recombination hot spots, suggesting they are not static and capable of some degree of plasticity.

Given that specific loci are repeatedly targeted in the development of functional traits, we propose that greater attention should be paid to the plasticity of molecular mechanisms that shape the genome. The biased distribution of crossover events—particularly those mediated by recombination hotspots sensitive to environmental conditions—suggests that the genome is not a passive substrate but a dynamic landscape influenced by ecological context. This has profound implications for evolutionary genetics, especially when considering how de novo mutations arise with surprising regularity in particular genes or regulatory regions. The probability of independently targeting the same gene codons across lineages by chance alone is vanishingly small, challenging the sufficiency of classical models based solely on stochastic mutation and selection. These patterns call for a deeper exploration of the molecular intermediaries that may channel variation in non-random ways.

### 1.4. Revisiting Mutational Bias Through an Epigenetic Lens

**Figure 2:**
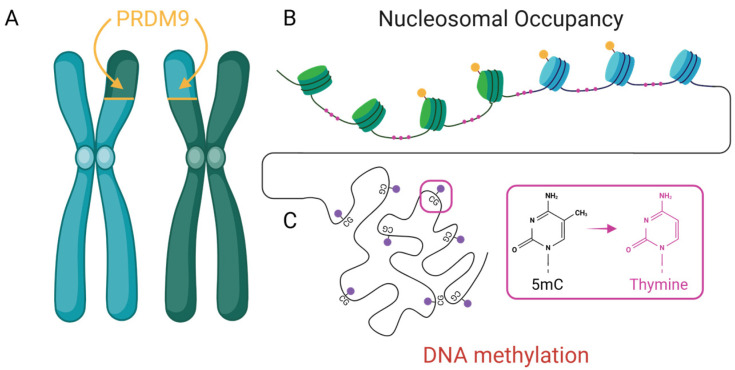
Three well-characterized epigenetic mechanisms contribute to non-random mutation and recombination. (**A**) PRDM9-directed histone modifications (yellow) define recombination hotspots as illustrated with green/blue nucleosome boundaries. (**B**) Nucleosome occupancy influences DNA accessibility and repair with exposed linker DNA subject to mutational bias (pink). and (**C**) DNA methylation at CpG dinucleotides (red) and its deamination can lead to transversions (pink).

Epigenetic regulation encompasses a diverse array of chromatin-based mechanisms that influence gene function without altering the underlying DNA sequence. Among the best-characterized are DNA methylation, histone modifications, and nucleosome positioning—all of which play critical roles in regulating gene expression, chromatin accessibility, and genome stability. While traditionally studied for their roles in transcriptional control, accumulating evidence suggests these mechanisms also shape where and how mutations and recombination events occur. In this section, we focus on three well-established epigenetic processes with particular relevance to mutational and recombinational bias (Figure 2): (1) DNA methylation, which contributes to context-dependent mutation rates and the evolution of regulatory elements; (2) PRDM9-mediated histone modification, which directs the placement of recombination hotspots and impacts lineage-specific genome evolution; and (3) nucleosome occupancy, which modulates mutation likelihood through its influence on DNA accessibility and repair. These examples are not intended to be an exhaustive list, but rather a representative sampling of plastic molecular processes through which the epigenome can bias mutation and recombination. Each provides a distinct but complementary lens for understanding how environmentally responsive and developmentally regulated chromatin features may channel genetic variation toward particular regions of the genome. Taken together, they illustrate how epigenetic architecture contributes to the direction, repeatability, and context-dependence of genome evolution, highlighting a broader need to integrate epigenomics into models of evolutionary change.

#### 1.4.1. Silent Marks, Loud Consequences: DNA Methylation and Mutation Bias

DNA methylation is a conserved epigenetic mark involved in transcriptional repression, transposon silencing, and imprinting. Beyond these regulatory roles, methylation—especially at CpG dinucleotides—has significant evolutionary consequences. Methylated cytosines are chemically unstable and prone to spontaneous deamination, leading to C → T transitions at rates 10 to 50 times higher than those at unmethylated sites [25] (Figure 2). This makes CpG sites disproportionately represented in mutation datasets across taxa. Traditionally viewed as a passive byproduct of base chemistry, methylation is emerging as a dynamic influence on mutational landscapes. Methylation patterns vary across tissues [58,59], developmental stages [60,61], and in response to environmental stressors [62], introducing spatiotemporal variability in mutational risk. CpG-rich regulatory regions (such as promoters and enhancers) are particularly affected, as both their functional importance and methylation density render them mutation-prone.

Notably, the deamination of methylated CpGs frequently gives rise to new transcription factor binding sites, particularly in regulatory regions [63]. These mutations can rewire cis-regulatory architecture and gene expression without altering coding sequences, potentially accelerating regulatory divergence or enabling parallel changes in distinct lineages. For example, CpG → TpG transitions in the MC1R gene have repeatedly contributed to melanism in vertebrates, including mice, birds, and lizards, often through regulatory or coding changes that impact pigmentation phenotypes [64,65]. Similar CpG deamination events have also been demonstrated to generate novel transcription factor binding sites in mammalian genomes, providing a potential mechanism for rapid regulatory evolution [63]. In addition to altering cis-regulatory potential, CpG mutations may further disrupt recognition by 5-methylcytosine (5mC) reader proteins, such as methyl-CpG-binding domain (MBD) proteins [66,67], which help translate methylation marks into transcriptional repression [68]. Although the MBD is broadly conserved [69,70], the proteins themselves show substantial lineage-specific variation, and their contribution to gene regulation and trait evolution remains poorly understood outside of mammalian systems. The evolutionary implications of 5mC readers represent an important, as yet understudied, frontier in comparative epigenetics [71].

These dynamics are evident across a wide range of taxa. In *Arabidopsis thaliana*, DNA methylation has been shown to shape the majority of genomic variance, with stress-induced methylation elevating mutation rates in specific genomic regions [72]. In humans, recurrent CpG mutations often arise in methylated regulatory elements, many associated with disease loci [73,74,75,76], highlighting methylation’s role in both genomic instability and evolutionary innovation. Importantly, these mutations are not limited to somatic tissues: studies have shown that methylated CpGs are enriched among germline de novo mutations transmitted between generations [74]. In contrast, in somatic cells, they contribute to mutational signatures characteristic of aging and cancer [76]. This dual presence across the soma/germline boundary emphasizes methylation’s broad impact on genome evolution, from intergenerational inheritance to disease-associated mosaicism. Together, these findings position DNA methylation not only as a regulator of gene expression but as an epigenetically encoded source of mutation bias capable of shaping evolutionary paths in ways that may be predictable, repeatable, and lineage-specific.

#### 1.4.2. PRDM9: The Cartographer of the Recombination Landscape

One of the most well-studied examples of an epigenetic regulator shaping mutational and recombination landscapes is the vertebrate gene PRDM9. PRDM9 has been deeply implicated in the formation of recombination hotspots in rodents [77,78], birds [79], and fish [80]. This gene codes for a histone methyltransferase that trimethylates histone H3 at lysine 4 (H3K4me3) and lysine 36 (H3K36me3). These modifications create an open chromatin structure conducive to double-strand break (DSB) formation, enhancing the likelihood of recombination at hotspot regions [81]. By creating a vulnerable epigenetic landscape, PRDM9 allows the requisite molecular machinery to access and process chromatin more efficiently during meiosis [82]. Importantly, this activity aligns meiotic recombination with functionally relevant genomic features such as promoters and CpG islands, regions often enriched in regulatory elements [49]. This non-random targeting suggests that recombination is not solely a stochastic process but one that may be epigenetically guided toward regions of evolutionary relevance, providing a plausible mechanism by which epigenetic factors contribute to convergent evolutionary outcomes.

Further evidence of PRDM9’s evolutionary significance comes from studies in murine species showing that the rapid evolutionary turnover of PRDM9 binding sites leads to distinct recombination landscapes between closely related subspecies [83]. This turnover results in asymmetric DSB formation in hybrids, disrupting meiosis and contributing to reproductive isolation. These findings suggest that PRDM9-mediated recombination is not only a dynamic and lineage-specific process but one that can influence evolutionary trajectories by shaping the potential for genetic exchange and hybrid fertility. Within humans, allelic variants of PRDM9 show their own distinct mutational signatures. For example, certain PRDM9 alleles common in African individuals activate a unique set of recombination hotspots that are largely inactive in individuals of European ancestry [84,85]. Similarly, PRDM9 can have reciprocal effects on its own coding sequence, leading to de novo additional variants capable of reshaping its own mutational landscape [86]. Beyond the PRDM9 locus itself, its long-term activity contributes to the erosion and evolutionary turnover of recombination hotspots, reflecting a dynamic and self-modifying role in shaping the recombination landscape across generations [87]. These findings highlight how a histone-modifying enzyme, like PRDM9, can introduce population-specific biases in the recombination landscape, reinforcing the idea that meiotic recombination is not a uniform or random process, but one that may be evolutionarily optimized in response to environmental, demographic, or genomic contexts. Such variation suggests that epigenetic processes can shape lineage-specific pathways to trait evolution, with implications for how mutational bias contributes to convergent outcomes across diverse populations.

#### 1.4.3. Tightly Wound, Lightly Wounded: Nucleosomal Armor in Mutation Bias

Nucleosomes, the fundamental structural units of chromatin, consist of approximately 147 base pairs of DNA wound around an octamer of histone proteins. While their primary function is to compact the genome, nucleosomes also regulate the accessibility of DNA to core cellular processes such as transcription, replication, and repair [88,89]. Their positioning is far from random, shaped by intrinsic DNA sequence preferences, histone post-translational modifications, and chromatin remodeling factors. This positioning defines regions of high or low accessibility within the genome, creating an architecture that is both stable and dynamic, capable of responding to developmental cues and environmental stimuli [89,90,91]. But beyond regulating access to DNA, could this dynamic chromatin architecture also influence where mutations occur and ultimately how genomes evolve?

Chromatin structure has direct implications for the mutational landscape of the genome. DNA tightly bound within nucleosomes is generally less exposed to mutagens and exhibits lower mutation rates compared to linker DNA or nucleosome-depleted regions [91,92,93,94]. For example, nucleosomal–DNA interactions can shield DNA from oxidative mutations [94] and radiation-induced damage [95]. However, once damage occurs, nucleosome-associated DNA is often repaired more slowly, giving rise to distinctive mutational signatures [96]. One striking example is the ~10 base pair periodicity in mutation frequency, which corresponds to the helical turns of DNA around the histone core [27]. This periodicity suggests that the physical interaction between DNA and histones modulates not just gene expression but also the likelihood and pattern of mutation. Since nucleosome occupancy is environmentally regulated and varies across cell types and developmental stages, it introduces a spatially patterned and responsive layer of mutational bias. Over evolutionary timescales, such biases may contribute to differential rates of mutation across genomic regions, affecting regulatory evolution and potentially predisposing certain loci to recurrent change [26]. Thus, nucleosome architecture serves not only as a scaffold for gene regulation but also as a mutational filter that may shape the direction and repeatability of evolutionary change.

The structural organization imposed by nucleosomes significantly shapes patterns of genome evolution. DNA wrapped within nucleosomes is less accessible to damage and mutates at lower rates, while exposed linker regions accumulate mutations more readily, creating localized hotspots for evolutionary change. This differential susceptibility influences regulatory evolution, as promoters and enhancers often lie in nucleosome-depleted regions that are more prone to sequence turnover. Such patterns may also bias parallel evolution by repeatedly exposing the same regulatory loci to mutation across independent lineages, increasing the likelihood of recurrent adaptive changes. Within coding regions, nucleosomes are frequently positioned over exons, where they may help preserve protein function by limiting mutational variability. On a broader scale, nucleosome occupancy patterns can highlight genomic regions under constraint or primed for innovation, offering insight into how chromatin structure helps channel the direction and repeatability of evolutionary change.

### 1.5. Epigenetic Bias and the Puzzle of Convergent Evolution

**Figure 3:**
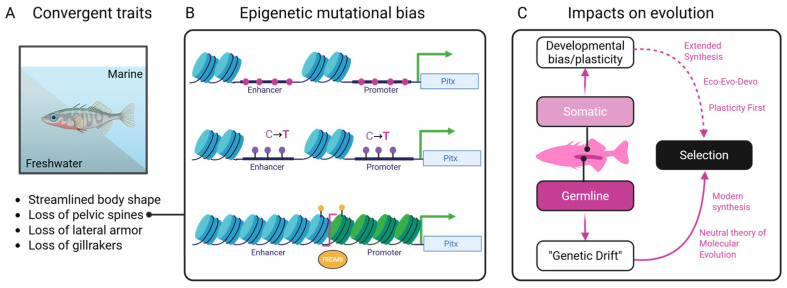
Conceptual synthesis showing how environmental pressures, epigenetic processes, and genetic mechanisms interact to shape convergent outcomes in stickleback fish. (**A**) Environmental inputs and their known biases on functional traits alter (**B**) chromatin states through DNA methylation, histone modification, and nucleosome remodeling of the Pitx gene leading to mutational biases (pink). (**C**) Classical genetic processes act upon this biased substrate leading to changes in traits that are subject to natural selection.

Convergent evolution, where similar traits evolve independently in distantly related taxa, poses a fundamental challenge to traditional evolutionary models. While shared selective pressures can account for adaptive similarity, the repeated targeting of the same genes or regulatory elements across lineages often defies explanation by chance alone. In many cases, these parallel changes occur despite the absence of shared ancestry or homologous genomic architecture [97]. This discrepancy prompts the need for additional mechanisms that constrain and bias evolutionary pathways (Figure 3). Epigenetic processes offer a compelling framework for explaining the repeatability of convergence. Because they shape which regions of the genome are accessible, mutable, and responsive to the environment, they may influence where mutations are more likely to occur. This insight is especially critical for understanding why certain loci are recurrently targeted in independent evolutionary events [65,97].

One illustrative example is the repeated evolution of pelvic reduction in freshwater stickleback fish. This phenotype has been traced to recurrent deletions in a regulatory enhancer of the Pitx1 gene [98,99]. These mutations occur in a tissue-specific enhancer that is epigenetically silenced in the pelvic tissue [99]. This suggests that its chromatin state may make it more vulnerable to mutational events such as deletions or transposon activity as evidenced from Pitx1 enhancer fragility [100]. Similarly, melanic pigmentation patterns in cichlids [101,102,103], lizards [104,105], birds [106,107], and mammals [64,65,108] often involve parallel targeted changes in genes like Mc1r, Agouti, or their regulatory elements. The repeated involvement of these loci implies that chromatin structure and accessibility may constrain which genes contribute to visible trait variation. The convergent loss of eyes in cave-dwelling organisms, including cavefish and subterranean mammals, presents another puzzle. Although natural selection clearly plays a role, the same genes (such as Oca2, Shh, and Pax6) are often independently disrupted in different lineages [109]. These loci, such as Pax6, are deeply involved in plastic responses to light exposure and are epigenetically regulated during early development. For instance, in Astyanax mexicanus, hypermethylation of Pax6 and other eye genes (triggered by environmental light exposure) silences their expression in early development without altering the DNA sequence, leading to eye degeneration in multiple cavefish populations [110,111]. If chromatin remodeling during eye regression renders these regions particularly fragile or mutation-prone, the epigenetic state could predispose them to convergent loss.

Parallelism of this sort extends beyond animals. In plants, traits like trichome density and flower symmetry have also evolved convergently through changes in shared transcriptional regulators. For example, the R2R3-MYB transcription factor family governs trichome initiation across flowering plants, with repeated use of homologous regulators such as GL1 in Arabidopsis and its functional analogs in cotton and tomato [112]. Similarly, bilateral floral symmetry has evolved multiple times via parallel redeployment of TCP and MYB modules, notably CYC and RAD-like genes, in both model and non-model angiosperms [113,114,115]. These examples reinforce the idea that a shared regulatory architecture may bias evolution toward certain genetic solutions, especially when developmental plasticity and modular transcriptional control intersect. Yet the question remains: why are the same gene networks repeatedly recruited during evolution? If their promoters are found in hypomethylated, nucleosome-depleted regions, they may be more prone to mutation or recombination, particularly under environmental stress. This hypothesis is supported by studies in Arabidopsis, where stress-induced methylation has been shown to elevate mutation rates in specific genomic regions [72].

### 1.6. Agency in the Making: Signaling, Epigenetics, and the Future of Convergence

Cells integrate environmental information through diverse signaling cascades that ultimately remodel the epigenome and influence phenotype. While mapping epigenetic states such as DNA methylation, histone modifications, and non-coding RNAs is now routine, the upstream processes that direct these changes remain comparatively undercharacterized. In most studies, epigenetic marks are treated as endpoints correlated with traits rather than as intermediates in a signaling chain that begins outside the nucleus [116]. This imbalance between outcome and process limits our ability to explain how environmental signals are translated into specific epigenomic programs that may bias adaptation and convergence.

Several pathways nonetheless provide proof of principle that such links exist. Steroid and nuclear hormone receptor signaling is one of the clearest cases: stress hormones and sex steroids bind intracellular receptors that move to the nucleus and recruit chromatin modifiers, reshaping transcriptional programs [117,118,119]. In the nervous system, experience-dependent dopamine and serotonin release activate intracellular cascades, including cAMP/PKA and ERK, that converge on CREB and lead to histone acetylation or methylation at plasticity-related genes [120,121]. Immune and inflammatory pathways follow a similar logic. Cytokine signaling activates NF-κB and STAT transcription factors that recruit chromatin remodelers and reprogram enhancers, while innate immune “training” produces durable histone modifications after exposure [122,123,124]. Nutritional and metabolic inputs also influence epigenetic states. One-carbon metabolism supplies methyl groups for DNA and histone methylation, as shown in the Agouti mouse model where maternal diet alters offspring coat color and obesity risk [108,125]. Microbiome-derived metabolites such as butyrate inhibit histone deacetylases and increase histone acetylation [126,127]. Even behavioral and social contexts can directly shape epigenomic states. Early-life maternal care alters chromatin at stress-axis genes in the hippocampus [117], and sensory experience in songbirds triggers calcium-dependent CREB activation and histone acetylation at genes involved in auditory learning [128].

These cases show that environment-to-epigenome signaling chains exist, yet they are often system-specific, fragmented across subfields, and rarely studied in the context of convergent evolution. Our ability to catalog epigenetic outcomes still far exceeds our mechanistic understanding of the signaling processes that generate them. Without greater clarity in mapping these upstream pathways, it remains difficult to construct a full agency-based framework in which cells and tissues are modeled as adaptive agents steering evolutionary trajectories toward recurrent solutions. Moving toward this framework should be a central goal for the field. By uncovering the repertoire of signaling pathways that connect environmental cues to epigenomic regulation, we will be better positioned to explain why evolution so often converges on similar molecular and phenotypic solutions. It is worth noting that this perspective echoes some of the earliest evolutionary ideas. Lamarck’s concept of the inheritance of acquired traits, although largely dismissed in its original form, anticipated the intuition that organisms might actively translate environmental experiences into heritable change. Modern epigenetics and the development of agent-based frameworks do not vindicate Lamarck’s mechanism. Instead, they revive the broader idea that the environment leaves structured imprints on heredity, now framed in a rigorously testable form.

#### Prisms, Not Pipelines: A Spectrum of Convergent Mechanisms

Beyond the focal mechanisms we have highlighted, a wide array of additional genomic processes contribute to convergent evolution, often in ways that blur the boundaries between “genetic” and “epigenetic” regulation. For example, non-coding RNAs such as microRNAs can canalize developmental pathways, biasing gene expression toward repeatable outcomes across taxa; one well-studied case is the role of miR-1 and miR-133 in the convergent evolution of muscle phenotypes across vertebrates [129,130]. Other classes of ncRNAs, including long non-coding RNAs and RNA modifications that alter chromatin interactions, also provide layers of regulatory control with evolutionary consequences [131,132]. These diverse RNA-mediated processes reveal how epigenetic regulation can constrain or bias mutational outcomes, thereby facilitating repeatable phenotypic trajectories.

Similarly, transposable elements have repeatedly been co-opted as regulatory modules—such as in mammalian placental evolution—where formerly mobile sequences now serve as enhancers controlling key developmental genes [20,133]. Structural rearrangements including inversions and deletions also influence convergence: inversions in Heliconius butterflies have preserved adaptive wing-patterning haplotypes that recur in mimicry rings [134], while deletions in the Pitx1 enhancer underlie repeated pelvic reduction in sticklebacks [98]. Viral incursions likewise represent an underappreciated force of epigenetic modification, with endogenous retroviruses in particular leaving regulatory imprints that shape lineage-specific traits [135,136]. Together, these mechanisms emphasize how diverse forms of genomic restructuring interact with epigenetic control to guide parallel outcomes.

Each of these processes is accompanied by its own epigenetic complement—transposon silencing through methylation, RNA-based regulatory loops, and chromatin remodeling at rearrangement breakpoints—that further modulates their evolutionary outcomes [21,137]. In addition, host-associated microbiomes have been shown to influence epigenomic regulation and shape adaptive traits, occasionally producing parallel outcomes across taxa [138], although a full treatment of this topic lies outside the scope of the present review. Taken together, these examples underscore that our focus on three epigenetic mechanisms represents only one prism through which to view convergence. Just as white light refracts into many colors, the study of convergence refracts into many molecular layers, each interacting with the others to channel repeatable evolutionary solutions. While genes, mutations, and structural changes provide a simple explanatory frame, adding the epigenetic lens reveals a far richer spectrum of mechanisms shaping how similar traits evolve again and again across the tree of life.

## 2. Epigenomic Structure as a Substrate for Convergent Evolution

Despite these examples, current models of convergence do not formally incorporate epigenomic structure. Many fail to account for the dynamic nature of chromatin during development or the role of environmentally induced plasticity in shaping long-term evolutionary outcomes. For example, no existing model explains why developmental enhancers—which are often methylated and subject to chromatin remodeling—are repeatedly co-opted in trait convergence. The structuring of the genome by epigenetic mechanisms provides a non-random substrate upon which mutation and selection can act. By influencing which genomic regions are accessible to change—based on chromatin state, DNA methylation, and environmental responsiveness—epigenetic processes offer a potential explanation for why evolution sometimes takes similar routes across disparate lineages. This perspective not only enriches our understanding of convergent evolution but also expands the evolutionary framework to include the dynamic and context-sensitive architecture of the genome itself.

## Data Availability

No new data were created or analyzed in this study. Data sharing is not applicable to this article.
